# Evolution of the nuclear ribosomal DNA intergenic spacer in four species of the *Daphnia pulex *complex

**DOI:** 10.1186/1471-2156-12-13

**Published:** 2011-01-24

**Authors:** Cheryl D Ambrose, Teresa J Crease

**Affiliations:** 1 Department of Integrative Biology, University of Guelph, Guelph, ON N1G2W1, Canada

## Abstract

**Background:**

Concerted evolution refers to the pattern in which copies of multigene families show high intraspecific sequence homogeneity but high interspecific sequence diversity. Sequence homogeneity of these copies depends on relative rates of mutation and recombination, including gene conversion and unequal crossing over, between misaligned copies. The internally repetitive intergenic spacer (IGS) is located between the genes for the 28S and 18S ribosomal RNAs. To identify patterns of recombination and/or homogenization within IGS repeat arrays, and to identify regions of the IGS that are under functional constraint, we analyzed 13 complete IGS sequences from 10 individuals representing four species in the *Daphnia pulex *complex.

**Results:**

Gene conversion and unequal crossing over between misaligned IGS repeats generates variation in copy number between arrays, as has been observed in previous studies. Moreover, terminal repeats are rarely involved in these events. Despite the occurrence of recombination, orthologous repeats in different species are more similar to one another than are paralogous repeats within species that diverged less than 4 million years ago. Patterns consistent with concerted evolution of these repeats were observed between species that diverged 8-10 million years ago. Sequence homogeneity varies along the IGS; the most homogeneous regions are downstream of the 28S rRNA gene and in the region containing the core promoter. The inadvertent inclusion of interspecific hybrids in our analysis uncovered evidence of both inter- and intrachromosomal recombination in the nonrepetitive regions of the IGS.

**Conclusions:**

Our analysis of variation in ribosomal IGS from *Daphnia *shows that levels of homogeneity within and between species result from the interaction between rates of recombination and selective constraint. Consequently, different regions of the IGS are on substantially different evolutionary trajectories.

## Background

We expect duplicated gene copies to accumulate mutations independently of one another, which results in greater sequence diversity among paralogs than among orthologs. However, in some multigene families (MGF), including ribosomal DNA (rDNA), tandemly arrayed paralogs are more similar to each other than they are to orthologs in closely related species. This pattern is referred to as concerted evolution [1], and Arnheim [2] invoked gene conversion and unequal crossing over between misaligned members of the gene family to explain it. Thus, the degree of sequence homogeneity within a MGF will depend on the relative rate of mutation and recombination between misaligned copies on homologous and nonhomologous chromosomes.

The ubiquity and high degree of interspecific sequence conservation of the genes encoding ribosomal RNA (rRNA) makes them a valuable system for studying MGF evolution. Tandem copies of these coding sequences alternate with the less-conserved intergenic spacer (IGS) and internal transcribed spacer (ITS) to form a complete ribosomal DNA (rDNA) unit. In many species, the IGS is internally repetitive, and contains one or more arrays of repeats with elements that may be involved in transcription regulation (*Drosophila *[3], *Xenopus *[4], *Arabidopsis *[5], rat [6], mouse [7], *Acanthamoeba *[8]). Furthermore, these elements are involved in chromosomal pairing in *Drosophila *[9]. The iterative nature of rDNA, the homogeneity of its copies and the regulatory functions played by the IGS suggest that recombination in the form of gene conversion and unequal crossover is frequent, and may be the result of DNA repair mechanisms influenced by protein/DNA interactions within it [10,11].

In a study of IGS repeat array variation in three populations of *Daphnia pulex*, Crease [12] reported greater similarity between orthologous copies of IGS repeats than between paralogous copies in the same repeat array. Hayworth [13] described similar results in a study of IGS variation in six species of *Arabidopsis*, although patterns typical of concerted evolution emerged as divergence times between species increased. In a previous study, we [14] reported few differences in IGS array organization and repeat sequences between the closely related species, *Daphnia pulicaria *and North American *Daphnia pulex *(*D. pulex*NA), but we observed clear differences between *Daphnia parvula *and *Daphnia obtusa*, which are in a different species complex than *D. pulex *or *D. pulicaria *and diverged from them on the order of 50-90 million years ago [15]. However, we also observed differences between an IGS array in European *D. pulex *(*D. pulex*E) and an array from *D. pulicaria *and *D. pulex*NA, all three of which are members of the *D. pulex species *complex.

In this study, we focus on evolutionary changes across the IGS, including the repeat arrays, by analyzing complete IGS sequences from representatives of four species in the *D. pulex *complex: *D. pulex*E, *D. pulex*NA, *D. pulicaria*, and *D. tenebrosa *(Table 1). *Daphnia pulex*NA is the dominant *Daphnia *species in ephemeral ponds that lack fish across North America while *D. pulicaria *has approximately the same geographic distribution but has invaded permanent lakes that contain fish. *Daphnia tenebrosa *is an Arctic endemic that lives in permanent ponds and lakes [16], and *D. pulex*E inhabits ponds in the temperate regions of Europe [17]. The divergence time between *D. tenebrosa *and *D. pulex*NA or *D. pulicaria *(~4-5 million years) is about half the divergence time between *D. pulex*E and the latter two species (~8-10 million years, [17]). Our objectives are to 1) identify patterns of recombination and/or homogenisation within rDNA repeat arrays, 2) estimate the divergence time at which repeats become more similar within species than between, and 3) identify regions of the IGS that may be experiencing functional constraint.

**Table 1:** *Daphnia *individuals included in this study.

Species	**Individual/IGS sequence**^**1**^	**GenBank Acc. No**.	Collection Locale	**Source**^**2**^
*D. pulex*NA	DpxNA1	EU595551	Champaign Co. Illinois, USA	T.J. Crease
	DpxNA2	EU595552	Vermillion Co. Illinois, USA	T.J. Crease
	DpxNA3	L07948^3^	Warren Co. Indiana USA	T.J. Crease
*D. pulex*E	DpxE1a	EU595553	Kola, Western Siberia	L.J. Weider
	DpxE1b	EU595554		
	DpxE2a	EU595555	Malente, Germany	L.J. Weider
	DpxE2b	EU595556		
	DpxE3a	EU595557	Preetz, Germany	L.J. Weider
	DpxE3b	EU595558		
*D. pulicaria*	Dpc1	EU595559	Greenland	L.J. Weider
	Dpc2	EU595560	Greenland	L.J. Weider
	Dpc3	EU595561	Humboldt Lake, Saskatchewan, Canada	T.J. Crease
*D. tenebrosa*	Dten	EU595562	Svalbaard, Norway	L.J. Weider

1. Individuals of the same species are numbered from 1 to 3. The two IGS sequences from each *D. pulex*E individual are designated "a" and "b".

2. Source refers to the person who originally collected the *Daphnia *and isolated genomic DNA from the parthenogenetic offspring of each wild-caught female.

3. This sequence was obtained from GenBank.

## Results

### IGS sequence variation

**Figure 1:**
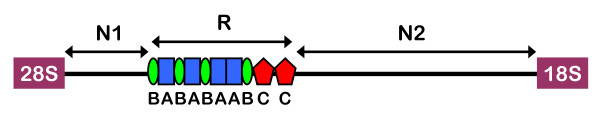
**Typical rDNA IGS from species in the *Daphnia pulex *complex**. 28S = 28S rRNA gene. 18S = 18S rRNA gene. N1 = nonrepetitive region 1. N2 = nonrepetitive region 2. R = repetitive mid section composed of A (blue), B (green), and C (red) repeats. N2 contains the external transcribed spacer (ETS).

We sequenced 13 complete IGS including one from each of three *D. pulex*NA (DpxNA1, DpxNA2, DpxNA3), three *D. pulicaria *(Dpc1, Dpc2, Dpc3) and one *D. tenebrosa *(Dten) individual. We sequenced two complete IGS from each of three *D. pulex*E individuals (DpxE1a, DpxE1b, DpxE2a, DpxE2b, DpxE3a and DpxE3b). Each individual was sampled from a different population (Table 1). We partitioned each IGS sequence into three regions: N1, the nonrepetitive segment located immediately downstream of the 28S rRNA coding region; R, the repetitive midsection; and N2, the nonrepetitive segment located downstream of the repeat arrays and 5' to the 18S rRNA coding region (Figure 1). The tandem arrays in the R-region were further dissected into repeat types A, B, and C.

**Figure 2:**
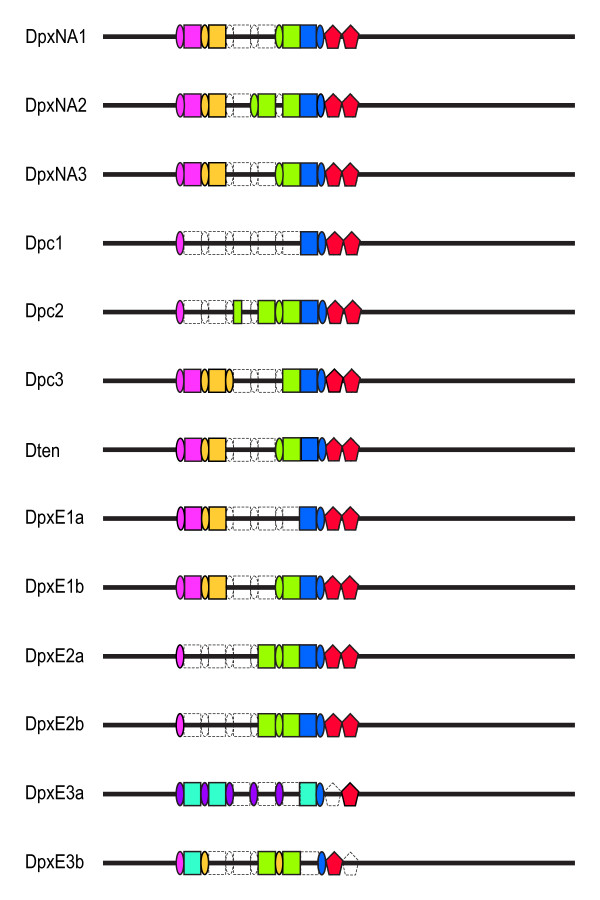
**Diagram of complete IGS sequences from representatives of four species in the *Daphnia pulex *complex**. Dpc = *D. pulicaria*, DpxE = European *D. pulex*, DpxNA = North American *D. pulex*, Dten = *D. tenebrosa*. A repeats are represented by rectangles, B repeats by ovals, and C repeats by pentagons. Repeats that cluster with one another in Neighbor-joining dendrograms are indicated by the same color. Open shapes bounded by dashed lines indicate putative deletions. Nonrepetitive regions upstream and downstream of the repeats are shown with thick black lines.

Length variation among the IGS sequences is as high as 20% and can be attributed to the presence of indels in the nonrepetitive regions (N1 and N2), and variable numbers of the three repeat types (A, B, and C) in the repetitive region, which begins between nt 659 (DpxNA3) and nt 797 (Dpc1), downstream of the 28S rRNA coding region (Figure 2, Additional file 1: Structure of complete IGS sequences). The three DpxNA IGS sequences have a 107 nt deletion beginning at nt 493 in the multiple alignment that is not present in any of the other species, and is not limited to these three populations (Crease TJ, unpublished data). Region N2 ranges from 2492 nt (DpxE1b) to 2843 nt (DpxE3a) in length.

**Figure 3:**
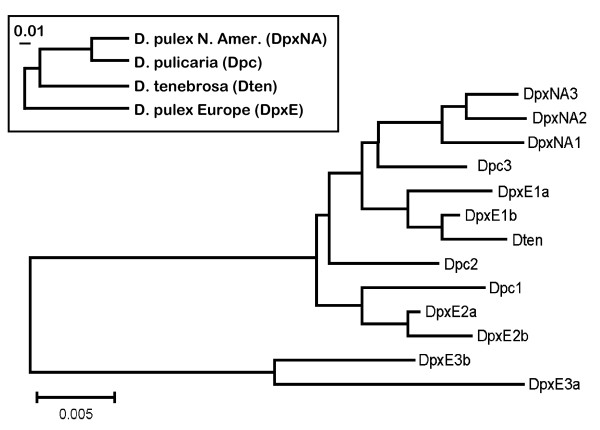
**Unrooted Neighbour-joining dendrogram of complete IGS sequences from representatives of four species in the *Daphnia pulex *complex**. The inset shows a phylogeny of these species based on the mitochondrial *ND5 *gene [17]. Dpc = *D. pulicaria*, DpxE = European *D. pulex*, DpxNA = North American *D. pulex*, Dten = *D. tenebrosa*.

**Figure 4:**
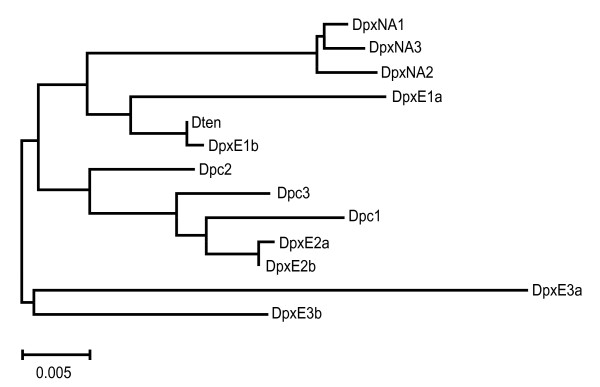
**Unrooted Neighbour-joining dendrogram of the IGS N1 region from representatives of four species in the *Daphnia pulex *complex**. Dpc = *D. pulicaria*, DpxE = European *D. pulex*, DpxNA = North American *D. pulex*, Dten = *D. tenebrosa*.

**Figure 5:**
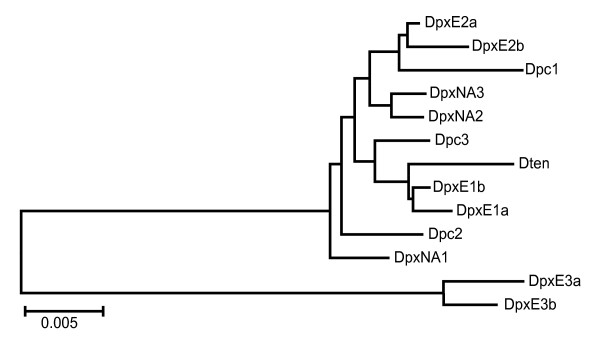
**Unrooted Neighbour-joining dendrogram of the IGS N2 region from representatives of four species in the *Daphnia pulex *complex**. Dpc = *D. pulicaria*, DpxE = European *D. pulex*, DpxNA = North American *D. pulex*, Dten = *D. tenebrosa*.

Neighbor-joining (NJ) trees based on the complete condensed (see methods) IGS, and the N1 and N2 regions (Figures 3, 4 and 5 ) show that the sequences from DpxE3 do form the sister group to the remaining sequences, as expected from phylogenies of the *D. pulex *complex based on mtDNA [17]. However, the DpxE1a/b and DpxE2a/b sequences do not cluster with them in any of these trees. In addition, Dten is not the sister group to a DpxNA+Dpc cluster, as expected based on the mtDNA phylogeny [17]. Indeed, the only consistent features of the three trees is the sister group relationship of the DpxE3a/b sequences to all the others, and the occurrence of two groups consisting of [Dpc1+DpxE2a/b] and [Dten+DpxE1a/b].

Mean sequence divergence (p-distance) in the complete condensed IGS and nonrepetitive regions is slightly higher between species than within them, with the highest values occurring in the N1 region (Table 2). Mean values of intra- and interspecific sequence divergence in the N1 and N2 regions are similar to those obtained for the complete IGS. The AMOVA results show that over half of the variation in complete IGS and N1 sequences occurs among species, but all the variation in N2 sequences occurs within species, although this value is not significant (Table 3). This result is consistent with the fact that N2 sequences show less clustering by species (Figure 5) than do the full IGS or N1 sequences (Figure 3, 4). For example, intraspecific sequence divergence is always lowest in DpxNA (Table 2), but all three sequences from this species do not cluster together in the NJ tree based on N2 sequences (Figure 5).

**Table 2:** Pairwise sequence divergence (PSD) between IGS sequences from ten individuals representing four species in the *Daphnia pulex *complex.

Region	Species	*D. pulex*NA	*D. pulicaria*	*D. tenebrosa*	*D. pulex*E
complete IGS^1^	*D. pulexNA*	0.009 [0.001]^2^			
	*D. pulicaria*	0.019 [0.002]	0.018 [0.002]		
	*D. tenebrosa*	0.017 [0.002]	0.019 [0.002]	n/c ^3^	
	*D. pulexE*	0.033 [0.002]	0.029 [0.001]	0.029 [0.002]	0.038 [0.002]
	Intraspecific mean PSD		0.022 [0.009]		
	Interspecific mean PSD		0.024 [0.003]		
N1^4^	*D. pulexNA*	0.006 [0.002]			
	*D. pulicaria*	0.043 [0.007]	0.023 [0.004]		
	*D. tenebrosa*	0.028 [0.006]	0.029 [0.005]	n/c	
	*D. pulexE*	0.043 [0.005]	0.034 [0.003]	0.030 [0.004]	0.043 [0.004]
	Intraspecific mean PSD		0.024 [0.011]		
	Interspecific mean PSD		0.035 [0.003]		
N2^5^	*D. pulexNA*	0.008 [0.001]			
	*D. pulicaria*	0.011 [0.001]	0.015 [0.002]		
	*D. tenebrosa*	0.015 [0.002]	0.017 [0.002]	n/c	
	*D. pulexE*	0.026 [0.002]	0.028 [0.002]	0.029 [0.002]	0.035 [0.002]
	Intraspecific mean PSD		0.019 [0.008]		
	Interspecific mean PSD		0.021 [0.003]		
A-repeat	*D. pulexNA*	0.068 [0.011]			
	*D. pulicaria*	0.081 [0.010]	0.101 [0.013]		
	*D. tenebrosa*	0.063 [0.009]	0.081 [0.010]	0.070 [0.012]	
	*D. pulexE*	0.082 [0.010]	0.096 [0.011]	0.079 [0.010]	0.093 [0.011]
	Intraspecific mean PSD		0.083 [0.008]		
	Interspecific mean PSD		0.080 [0.004]		
B-repeat	*D. pulexNA*	0.158 [0.023]			
	*D. pulicaria*	0.158 [0.021]	0.176 [0.025]		
	*D. tenebrosa*	0.143 [0.020]	0.154 [0.021]	0.184 [0.028]	
	*D. pulexE*	0.160 [0.021]	0.161 [0.021]	0.156 [0.021]	0.162 [0.022]
	Intraspecific mean PSD		0.170 [0.006]		
	Interspecific mean PSD		0.155 [0.003]		
C-repeat	*D. pulexNA*	0.036 [0.010]			
	*D. pulicaria*	0.032 [0.008]	0.036 [0.009]		
	*D. tenebrosa*	0.033 [0.009]	0.033 [0.008]	0.039 [0.010]	
	*D. pulexE*	0.033 [0.008]	0.033 [0.008]	0.030 [0.008]	0.052 [0.016]
	Intraspecific mean PSD		0.041 [0.004]		
	Interspecific mean PSD		0.032 [0.000]		

1. Repeat arrays have been replaced with one copy of the consensus for each repeat type in the complete IGS sequences.

2. Values in square brackets are standard errors.

3. Value not calculated as only one sequence was determined for *D. tenebrosa*.

4. N1 is the nonrepetitive region downstream of the 28S rRNA gene

5. N2 is the nonrepetitive region upstream of the 18S rRNA gene.

**Table 3:** Analysis of molecular variance in the rDNA IGS from ten individuals representing four species in the *Daphnia pulex *complex.

	Source of variation	**d.f. **^**2**^	Sum of squares	Variance components	Percentage of variation
entire IGS^1^	Among species	3	1901.24	162.88	50.82 *
	Within species	9	1418.83	157.65	49.18
	Total	12	3320.08	320.52	
N1	Among species	3	489.60	46.40	62.72 *
	Within species	9	248.17	27.57	37.28
	Total	12	737.770	73.97	
N2	Among species	3	282.91	-6.48	-6.07
	Within species	9	1019.17	113.24	106.07
	Total	12	1302.08	106.76	
A-repeat	Among species	2	34.90	-0.36	-1.71
	Among individuals within species	6	132.30	0.24	1.14
	Within individuals	32	671.90	20.107	100.57
	Total	40	839.10	20.88	
	FST = -0.01				
B-repeat	Among species	2	12.67	0.03	0.23
	Among individuals within species	10	63.39	-2.22	-18.15
	Within individuals	35	504.58	14.42	117.91
	Total	47	580.65	12.23	
C-repeat	Among species	2	3.68	-0.09	-1.84
	Among individuals within species	7	19.16	-1.64	-33.78
	Within individuals	14	92.00	6.57	135.61
	Total	23	114.83	4.85	

1. Repeat arrays have been replaced with one copy of the consensus for each repeat type in the complete IGS sequences.

2. degrees of freedom.

* p < 0.001.

**Figure 6:**
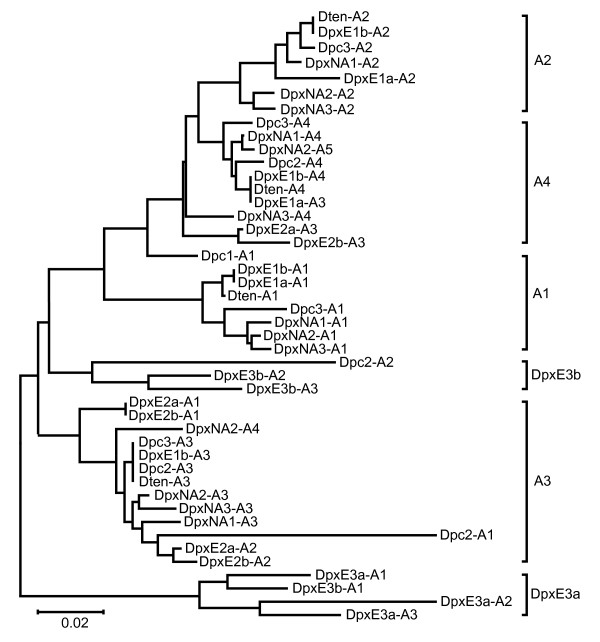
**Unrooted Neighbour-joining dendrogram of IGS A repeats from representatives of four species in the *Daphnia pulex *complex**. Dpc = *D. pulicaria*, DpxE = European *D. pulex*, DpxNA = North American *D. pulex*, Dten = *D. tenebrosa*.

In the repetitive region of the IGS, one to five copies of the A repeat, ranging from 184 to 222 nt in length, are interleaved with B repeats (Additional file 1, Figure 2). All sequence variation among A repeats is within individuals (Table 3). With the exception of two A repeats from DpxE3b and an anomalous Dpc2 A repeat, there is a tendency for repeats to cluster according to their position in the array (Figure 6). Mean sequence divergence between A repeats within clusters based on the NJ tree is 0.04 while that between sequences from different clusters is substantially higher at 0.125 (Table 4).

**Table 4:** Sequence divergence (p-distance) among IGS repeats in ten individuals representing four species in the *Daphnia pulex *species complex.

Repeat **type**^**1**^	Mean p-distance within clusters	Range of p-distance values	Mean p-distance between clusters	Range of p-distance values
A	0.040	0.023-0.079	0.125	0.054-0.176
B	0.027	0.005-0.080	0.168	0.045-0.286
C	0.013	0.011, 0.014	0.057	n/a

**Figure 7:**
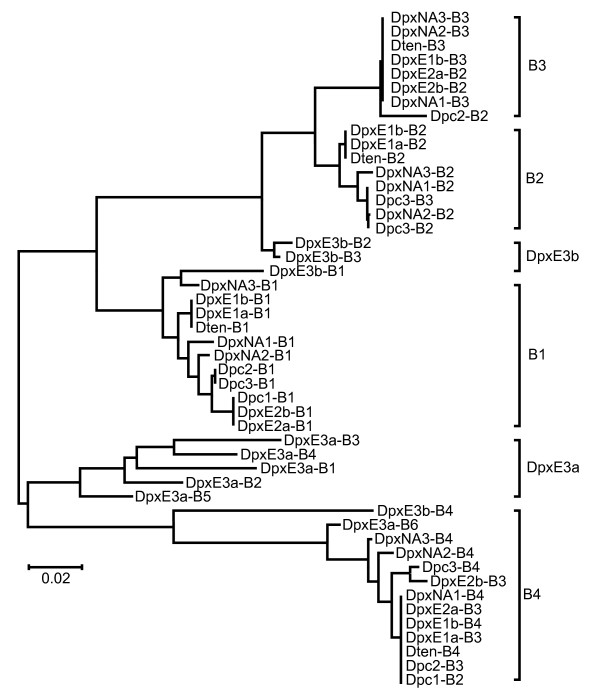
**Unrooted Neighbor-joining dendrogram of IGS B repeats from representatives of four species in the *Daphnia pulex *complex**. Dpc = *D. pulicaria*, DpxE = European *D. pulex*, DpxNA = North American *D. pulex*, Dten = *D. tenebrosa*.

**Figure 8:**
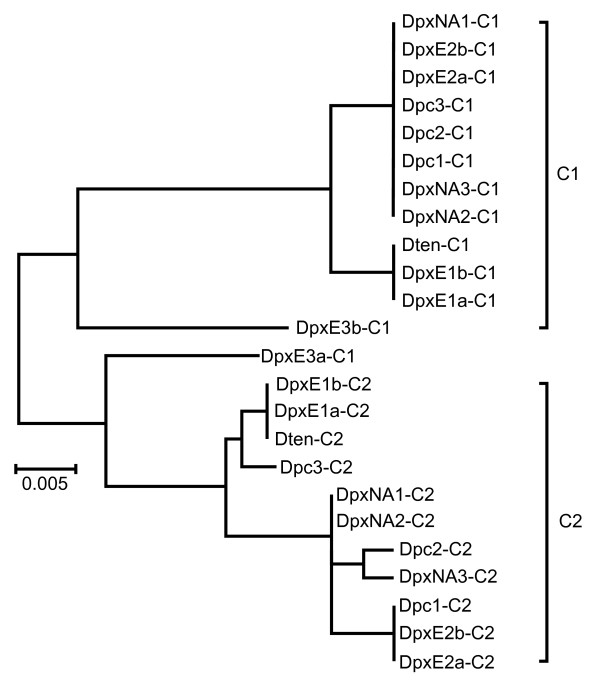
**Unrooted Neighbour-joining dendrogram of IGS C repeats from representatives of four species in the *Daphnia pulex *complex**. Dpc = *D. pulicaria*, DpxE = *D. pulex *Europe, DpxNA = *D. pulex *North America, Dten = *D. tenebrosa*.

1. Repeats of each type have been grouped by cluster based on the NJ trees in Figures 6, 7 and 8.

The number of B repeats per IGS ranges from two to six (Figure 2, Additional file 1). As with A repeats, all of the sequence variation is found within individuals (Table 3). Mean sequence divergence between sequences within the clusters based on the NJ tree (Figure 7) is 0.027 while that between sequences from different clusters is 0.168 (Table 4).

C repeats occur as two tandem copies in all but the two DpxE3 IGS sequences, which each contain a single C repeat (Figure 2). All sequence variation among C repeats is within individuals (Table 3) and repeats cluster by position (Figure 8). The single C repeat in DpxE3a groups with repeats in the second position, while the single C repeat in DpxE3b groups with those in the first position (Figure 8). Mean divergence between sequences within the clusters based on the NJ tree is 0.013 while that between sequences from different clusters is 0.057 (Table 4).

### Recombination

Although analysis of the complete condensed IGS using GARD partitioned it into five hypothetical nonrecombinant sections, the server's execution time limit per job was reached before the analysis was completed. Further analysis of N1 identified five hypothetical nonrecombinant sections, and although four such sections were identified in N2 (Table 5), the server once again timed out before the analysis of the alignment was complete. In the complete IGS, the first putative recombination breakpoint falls immediately upstream of the repeat array and the second is located within the A repeat consensus sequence, just upstream from the spacer promoter [18,19]. The position of the last two breakpoints coincides with two of the three breakpoints identified in the separate analysis of the N2 region. The third IGS breakpoint is about 150 nt downstream of the core promoter, and the fourth IGS breakpoint is located in the vicinity of a (GT)n microsatellite, a few hundred nt upstream of the 18S rRNA coding region.

**Table 5:** Pairwise sequence divergence (p-dist) among fragments within each section of the IGS from ten individuals representing four species in the *Daphnia pulex *complex.

**Section label**^**1**^	**Nucleotide Position 2**^**2**^	Features	**OAM **^**3 **^**p-dist**	**MIA **^**4 **^**p-dist (x)**	**MIR **^**5 **^**p-dist (y)**	p-dist ratio (x/y)
IGS	1 - 4339	entire IGS	0.028 [0.001]^6^	0.022	0.024	0.917
IGS-1	1 - 759	N1	0.037 [0.004]	0.026	0.035	0.743
IGS-2	759 - 1031	repeat consensus	0.078 [0.009]	0.078	0.083	0.940
IGS-3	1031 - 3150	N2 ~ 1340 core promoter ~3000	0.023 [0.002]	0.019	0.020	0.950
IGS-4	3150 - 3906		0.021 [0.003]	0.014	0.015	0.933
IGS-5	3906 - 4339		0.034 [0.005]	0.027	0.034	0.794

N1	1 - 841	entire N1 region	0.037 [0.004]	0.024	0.035	0.686
N1-1	1 - 170		0.001 [0.001]	0.001	0.001	1.0
N1-2	170 - 338		0.001 [0.001]	0.001	0.001	1.0
N1-3	338 - 503		0.057 [0.012]	0.040	0.053	0.755
N1-4	503 - 684		0.013 [0.004]	0.014	0.011	1.273
N1-5	684 - 841		0.036 [0.010]	0.017	0.034	0.500

N2	1-3022	entire N2 region	0.025 [0.001]	0.019	0.021	0.905
N2-1	1-657		0.019 [0.003]	0.016	0.017	0.941
N2-2	657-1515		0.023 [0.003]	0.019	0.023	0.826
N2-3	1515-2565	core promoter ~1660	0.024 [0.002]	0.017	0.017	1.0
N2-4	2565-3017		0.038 [0.005]	0.030	0.036	0.833

1. Fragments were defined by recombination analysis of multiple sequence alignments using the program GARD [44].

2. Nucleotide position in the multiple alignment.

3. Overall mean

4. Mean intraspecific

5. Mean interspecific.

6. Standard errors are given in square brackets.

**Figure 9:**
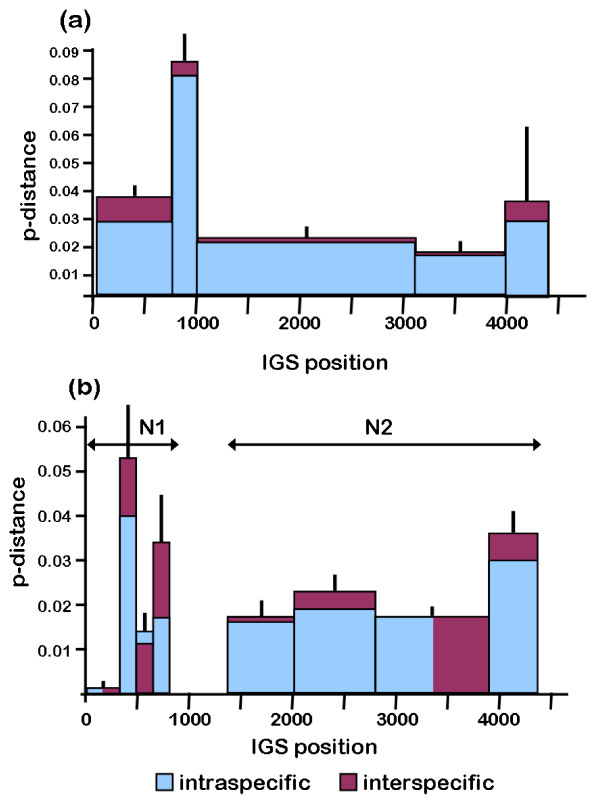
**Mean pairwise p-distance between IGS sequences from representatives of four species in the *Daphnia pulex *complex**. **(a) **The complete IGS. **(b) **Nonrepetitive regions, N1 and N2. Values are plotted for segments defined by recombination analysis of multiple sequence alignments using the program, GARD [44]. Segment 2 in the IGS corresponds to the R region, which contains the repeat arrays. Vertical black lines are standard errors of the overall mean p-distance (intraspecific and interspecific).

Mean inter- and intraspecific p-distances differ substantially among the regions identified by the GARD analysis (Table 5). The regions with the least variation are located just downstream of the 28S rRNA coding region (N1-1 and N1-2) while the regions with the highest variation are just downstream of that (N1-3) and in the A repeat consensus (IGS-2) (Table 5, Figure 9).

Analysis of complete IGS sequences using GENECONV identified 12 significant putative gene conversion tracts between ancestors of two sequences in the alignment. All 12 fragments are located within the N2 region, and range in length between 106 and 1620 nt (Additional file 2: Gene conversion analysis of complete IGS sequences). Three of these conversion tracts, including the longest one, are between IGS sequences from the same individual (DpxE1a × DpxE1b and DpxE3a × DpxE3b). Three other tracts are between sequences from different individuals of the same species (DpxE1b × DpxE2a; DpxE1b × DpxE2b; DpxE1a × DpxE3a). The six remaining exchanges involve interspecific gene conversion events; four tracts involve Dpc2: two with Dten and one each with DpxE1b and DpxE1a. Two tracts involve DpxE2b; one with Dten and one with DpxNA3 (Additional file 2). A separate analysis of the N1 region identified only two gene conversion tracts, both of which cover the same region, between DpxE3a and each of two DpxNA individuals (Additional file 3: Gene conversion analysis of the IGS N1 region.). In a similar analysis of N2 (Additional file 4: Gene conversion analysis of the IGS N2 region), four of the seven gene conversion tracts are interspecific, involving Dten with Dpc2 (2 tracts), DpxE3a and DpxE2b.

There is little statistical support for putative recombination breakpoints within the aligned consensus repeat sequences using the GARD algorithm. However, the GENECONV algorithm identified seven putative gene conversion tracts in the A repeat alignment and nine in the B repeat alignment (Additional files 5: Gene conversion analysis of the IGS A repeat region, Additional file 6: Gene conversion analysis of the IGS B repeat region). All putative gene conversion tracts in the B repeat alignment were between the fourth copy of the B repeat from DpxE3b and the second or third copies of B repeats from all other species. Two putative gene conversion tracts were identified between the C repeat from DpxE3a and the second C repeat copies from Dpc2 and DpxNA3 (Additional file 7: Gene conversion analysis of the IGS C repeat region).

## Discussion

### Hybridization

We found inconsistencies between the topology of NJ trees based on IGS sequences and the species phylogenies based on mtDNA sequences. The most plausible explanation for these differences is the introduction of allospecific nuclear DNA through hybridization between *D. pulex*E and both *D. tenebrosa *and *D. pulicaria*. The NJ tree of complete IGS sequences reveals that only the two DpxE3 sequences form a separate branch as predicted by the divergent mtDNA sequence of this individual. The IGS sequences of the other two *D. pulex*E individuals cluster with those from *D. pulicaria *or *D. tenebrosa*. While hybridization between *D. pulex*E and *D. pulicaria *or *D. tenebrosa *has not been documented in the literature, the divergence among their mitochondrial 12S rDNA sequences falls well below the 14% threshold for species which are known to hybridize [[15] and references within]. Colbourne and Hebert [15] note that the lack of evidence for hybridization between species with low levels of 12S rDNA sequence divergence involves taxa with allopatric distributions, which is generally thought to be the case for *D. pulex*E relative to the other species. However, *D. pulex*NA and *D. pulicaria *have been found in Europe [16,20] so opportunities for hybridization do exist.

The phylogenetic relationship among the four *Daphnia *species in this study, based on mtDNA sequences, is most closely reflected in the relationship among N1 sequences. We observed tight clustering of the three N1 sequences from *D. pulex*NA, while the N1 sequences of the three *D. pulicaria *individuals form a looser grouping. However, evidence for introgression is seen in four of the six DpxE N1 sequences. N1 sequences from DpxE1 cluster with the Dten N1 sequence, and N1 sequences from DpxE2 cluster with the Dpc N1 sequences.

The tree topologies of A and B repeats, which are interleaved with one another, are similar. Aside from the repeat sequences from DpxE3, major clusters are formed by orthologous rather than paralogous repeats for both A and B types. The occurrence of this structure in all but the most divergent species suggests that it has persisted for several million years, despite the occurrence of recombination between repeats (discussed below, [12]). Unfortunately, because all but one of the IGS arrays from *D. pulex*E appears to have been impacted by hybridization, it is not entirely clear if this position-specific pattern also occurs in this species. However, two observations suggest that it may. First, the only A repeats from different DpxE IGS sequences (DpxE3a-A1 and DpxE3b-A1) that cluster with one another in the NJ tree (Figure 6) are both in the same (first) position. Second, branch lengths between the A repeats in the DpxE3a array are more similar to branch lengths between array positions than within them in the other species (Figure 6).

All but the last of the six B repeats in the DpxE3a array cluster with one another, which is consistent with the pattern observed for A repeats. However, orthologous clustering of terminal F repeats was observed in the IGS of *Drosophila melanogaster *and *Dr. orena *[14]. Others have also reported the apparent escape from homogenization experienced by terminal repeats relative to interior paralogs [21-23].

With the exception of the three *D. pulex*E individuals, for which two complete IGS were sequenced, our data are limited to a single IGS sequence for each individual, and three IGS sequences per species in *D. pulex*NA and *D. pulicaria*. This, in combination with the introgression mentioned above, limits the confidence with which we are able to estimate the divergence time necessary for IGS sequences to appear more similar within than between species. However, divergence times between the species in this study, based on the mitochondrial genes [17], suggest that the threshold for detecting patterns consistent with concerted evolution for the complete IGS must be greater than 4 million years.

### Recombination in the IGS

We expect the hierarchically iterative nature of rDNA to facilitate recombination and homogenization at this locus. Indeed, our GARD and GENECONV analyses confirm that recombination occurs at multiple locations across the *Daphnia *IGS, including the repeats although these analyses do not identify recombination hotspots. The GARD algorithm identifies nonrecombinant segments rather than precise recombination break points and adopts the convention that breakpoints coincide with variable sites because breakpoints can only be resolved to the nearest variable site [24]. In fact, actual breakpoints may be located at invariant sites [25].

Although it is possible that some of the intraindividual recombination that we observed is due to template switching during PCR amplification, we used a long extension time and a total of 30 cycles. Thus, it seems unlikely that recombination during the PCR reaction is a substantial source of the variation we observed. Indeed, recombination among IGS repeats has been observed in sequences from *D. pulex *obtained by cloning directly from genomic DNA [12]. However, the frequency with which such artefacts occur could also be tested empirically by combining cloned divergent IGS sequences and amplifying them under our PCR conditions.

The copy number of A repeats, which contain a putative enhancer motif [18], ranges between one (Dpc1) and five (DpxNA2). Crease [12] reported that 18 of 21 arrays from seven *D. pulex*NA individuals contained four repeats, while the remaining three contained either five or six. This length variation is strong evidence that unequal crossing over is occurring between misaligned IGS repeats. Despite this, A and B repeats cluster by position in the array rather than species. This pattern was also observed by Luchetti et al. [26] in the IGS arrays of *Triops cancriformis*, which contain three copies of a ~200 nt repeat. In a previous study, we [14] found that the homogeneity of tandem and interleaved repeats increases as their number increases in arthropod IGS sequences. Thus, the rate of recombination in short arrays may be too low to fully homogenize the repeats. We also observed that duplication and deletion events rarely involve terminal repeats, which is consistent with the results of earlier work in plants. For example, Markos and Baldwin [27] found that interior repeats evolve in concert in *Lessingia spp*. (Compositae, Astereae), and Baldwin and Markos [28] found that sequence similarity of flanking repeats is higher between orthologs than paralogs in *Calycadenia *(Asteraceae).

Previous studies have suggested that intrachromosomal exchange (between sister chromatids) is more frequent than interchromosomal exchange (between homologues) in rDNA. For example, Crease [12] showed that intrachromosomal recombination is most likely responsible for patterns of sequence diversity within the IGS repeat arrays of *D. pulex*NA. Similarly, Schlötterer and Tautz [29] suggested that intrachromosomal exchange mechanisms are the most parsimonious explanation for the homogenization process in the ITS of *Drosophila melanogaster*. In contrast, our results suggest that many of the putative gene conversion tracts in the nonrepetitive regions of the IGS occurred between, rather than within, species (i.e. between homologous chromosomes in hybrids). This is consistent with the results of Polanco et al. [30] who showed that homogenization of the *Drosophila *IGS is the result of interchromosomal recombination. Our results do not exclude the possibility that intrachromosomal exchange occurs at an equal or even higher frequency than interchromosomal exchange. However, they do suggest that recombination within the IGS occurs during a phase in the cell cycle when homologous chromosomes are in close proximity, either following S phase during meiosis or when actively transcribed rRNA genes come together to form the nucleolus. Recombination can also occur between rDNA arrays on nonhomologous chromosomes, but *D. pulex *has only a single rDNA array per haploid genome (D. Tsuchiya, unpublished data). The number of rDNA arrays has not been determined for the other species, but they have similar genome sizes [31] and the same number of chromosomes (n = 12) as *D. pulex *[32]. Taken together, the above studies corroborate Polanco et al.'s [33] assertion that different regions within the rDNA unit follow different evolutionary trajectories.

### Conserved regions within the IGS

The exceptionally low sequence diversity in the first ~350 nt of N1 suggests that it undergoes homogenization along with the 28S rRNA gene. Liao [34] also reported that the homogenization of flanking regions in bacterial rRNA genes was the result of hitchhiking, or co-conversion with genic sequences. Moreover, the mean sequence divergence and the topology of NJ trees differs between N1 and N2, and from the repetitive region that connects them. This may be due to differences in the strength of natural selection acting on regulatory regions within the IGS, as well as the frequency with which recombination occurs between paralogous repeat copies whose sequences predispose them to frequent breakage and repair.

Because concerted evolution reduces mean intraspecific p-distance among the members of a MGF despite interspecific divergence, we would expect the ratio of mean intra- and interspecific p-distance (p-distance ratio) to be less than one and decrease with divergence time. On the other hand, if natural selection is constraining sequence divergence, then mean intra- and interspecific p-distance should be low and similar, especially among closely related taxa such as those included in this study. In this case, the p-distance ratio would remain close to one regardless of divergence time.

Although hybridization has blurred the species boundaries between individuals in this study, a comparison of mean p-distances within and between species does suggest that some of regions of the IGS may be under functional constraint. For example, the most conserved of the four N2 segments delimited by GARD breakpoints (N2-3), with a p-distance ratio of 1.0, is located between the putative core promoter and the breakpoint at nt 3900 in the full IGS, which may be the location of an rRNA processing site [35-37]. In contrast, the region that appears to be under the least functional constraint (N2-4, Table 5) is just downstream of this region and upstream of the 18S rRNA coding region, which is highly conserved both within and between species. This increase in both mean intra- and interspecific p-distance is also evident when mean p-distance is calculated after dividing the IGS into sequential 500 nt sections (data not shown).

As previously noted, the lowest overall sequence diversity occurs at the 3' end of the 28S rRNA coding region (N1-1 and N1-2). In contrast, the highest sequence diversity occurs just downstream of this region, in the middle section of N1 (N1-3), which includes a GA_n _dinucleotide repeat. The p-distance ratio is relatively low in this region (0.76, Table 5), but the lowest ratio (0.5) occurs in region N1-5, which is separated from N1-3 by the only region in the IGS (N1-4) where mean intraspecific divergence actually exceeds mean interspecific divergence (ratio = 1.27). The explanation for this pattern is unclear, but it should be noted that all of the regions in N1 are relatively short (151 - 181 nt). Further examination of this pattern will require analysis of species that diverged from a common ancestor at least 4 million years ago, and between which hybridization does not occur.

The region of the IGS with the highest mean intraspecific sequence divergence is the repeat region, although the p-distance ratio is also high at 0.94 (Table 5). This high level of diversity is primarily driven by differences between repeats in different positions in the array (Figure 3 and Figure 4). As suggested above, one explanation for this is low rates of recombination. However, it has also been suggested that this pattern may be maintained by natural selection despite the occurrence of recombination [12]. Indeed, the A repeats contain an ~27-nt putative TATA motif, which is highly conserved among all A repeats in this and the previous study [12]. This motif is also be found in the IGS repeats of other arthropods [14] and those containing the motif were found to be significantly more homogeneous than those without it in these taxa. These results suggest that selection is able to maintain homogeneity or diversity among functionally important repeat types regardless of the level of recombination among them [12].

## Conclusions

The occurrence of length variation in the IGS repeat array suggests that unequal crossing over occurs in this region, which is consistent with previous work. However, we also found evidence of interchromosomal gene conversion in the nonrepetitive regions of the IGS. Levels of sequence homogeneity vary across the IGS, due to the interaction between rates of recombination and selective constraint. The pattern of position-specific variation in *Daphnia *IGS repeat arrays likely predates speciation in the *D. pulex *species complex, and has persisted for millions of years. Although this pattern may be a function of low rates of recombination in short repeat arrays, the occurrence of a highly conserved TATA motif in the A repeats suggests that natural selection may also play a role in the maintenance of this pattern. The unintentional inclusion of hybrids in this study provided insight into evolutionary patterns within the IGS, but analysis of *D. pulex*E individuals that are not hybrids, as well as other sister species pairs in the genus *Daphnia*, is required to more precisely estimate the divergence time at which paralogous IGS repeats show evidence of concerted evolution.

## Methods

### Cloning and sequencing the IGS

Complete copies of the IGS were amplified from genomic DNA of *Daphnia *individuals using the Expand Long Template PCR System (Roche Diagnostics) and primers complimentary to highly conserved regions at the 3' end of the 28S rRNA gene (5' GTTTAGACCGTCGTGAGACAGGTTAG) and the 5' end of the 18S rRNA gene (5' TCAGGCTCCCTCTCCGG). The PCR conditions were as follows: 95°C for 5 min, followed by 10 cycles of 92°C for 30s, 60°C for 30s, and 68°C for 8 minutes. The next 20 cycles were 92°C for 30s, 60°C for 30s, and 68°C for 8 min plus 2s/cycle, ending with a final extension at 68°C for 7 minutes. Depending on the species, the major fragment generated in these amplifications ranged between ~4000 and 5500 nt, in agreement with earlier characterizations of the *D. pulex *IGS [12].

The PCR fragments were gel purified and cloned using the TOPO XL PCR cloning Kit (Invitrogen) according to manufacturer's recommendations. After screening plasmid clones for inserts of the expected size using gel electrophoresis, the presence of the 5' 18S and 3' 28S rRNA gene termini were confirmed by sequencing plasmid DNA with the M13 Forward and Reverse primers and the ABI Prism TaqFS dye terminator kit (Applied Biosystems). Sequences were resolved on an ABI 377 automated sequencer (Applied Biosystems). The Erase-a-base system (Promega) was used to generate nested deletion subclones from a single plasmid clone from each individual. Overlapping deletion subclones were sequenced and a contig sequence of each insert from each individual was created in Sequencher (Gene Codes). Additional clones from each species, and regions that were not well covered by deletion subclones were sequenced by primer-walking.

### IGS sequence analysis

We located the 5' end of the 18S rRNA gene and the 3' end of the 28S rRNA gene in each IGS sequence by consensus alignment with the homologous sequence from *D. pulex *[GenBank:AF014011] and *D. longicephala *[GenBank:AF346516] [38]. Repetitive regions in individual IGS sequences were located visually in Dotmatcher [39] using a window size of 25 and a threshold of 40. We estimated IGS repeat boundaries by performing 25 local self-alignments using the default settings in the local similarity program, SIM [40,41]. Finally, we refined putative IGS repeat boundaries by visual inspection of the aligned IGS repeat sequences in Multiple Sequence Alignment Program (MAP) [42] with default settings.

To facilitate alignments between individuals with different numbers and/or organization of A, B and C repeats, we aligned all copies of each repeat type for each IGS sequence and created a consensus repeat sequence. We then condensed the repeat array in each full IGS sequence by replacing it with one copy of the consensus for each repeat type and aligned the condensed IGS sequences using ClustalW [43]. The resulting alignment was adjusted by eye.

To incorporate a conservative estimate of the effects of indels on sequence divergence, we replaced the first position in each gap with a nucleotide that was unique to that position. We used the nucleotide p-distance algorithm in MEGA v3.1 [44] to calculate pairwise sequence divergence between entire condensed IGS sequences, the nonrepetitive regions (N1 and N2) and the repeat sequences. We used pairwise deletion of indels and assumed homogeneous patterns of nucleotide substitution among lineages and uniform mutation rates among sites for these analyses. MEGA was also used to construct Neighbor-joining dendrograms from the nucleotide p-distance matrices.

### Statistical analyses

We performed an Analysis of Molecular Variance (AMOVA) using Arlequin 3.0, [45] to partition the genetic variance in condensed IGS sequences within and between species. In addition, variation in each repeat type was partitioned within and between species. Levels of significance were based on 1023 random permutation replicates.

We used the program Genetic Algorithm Recombination Detection (GARD) [46] to look for evidence of recombination within each condensed IGS. In addition, we analyzed sequence alignments of the nonrepetitive N1 and N2 regions separately because the entire condensed IGS sequences caused the program to "time out" before all breakpoints had been identified. Goodness of fit was assessed by small sample Akaike Information Criterion (AIC) derived from a maximum likelihood model fit to each segment. Nucleotide substitution bias models were defined for each alignment using the model selection tool in GARD (general discrete rate variation with four rate classes, and the GARD detection method, which looks for multiple rather than a single breakpoint).

We also used the GENECONV v1.81 computer program [47] to find statistical evidence of putative gene conversion events between the ancestors of two sequences in an alignment. GENECONV identifies global inner fragments that contain evidence of a possible gene conversion event between ancestors of two sequences in the alignment. It also identifies outer fragments, which contain evidence of putative gene conversion events that may have originated from outside of the alignment, or from within the alignment, but with evidence of the source destroyed by later mutation or gene conversion [48].

## Abbreviations

bp: base pair; ETS: external transcribed spacer; IGS: intergenic spacer; ITS: internal transcribe spacer; MGF: multigene family; N1: nonrepetitive region 1; N2: nonrepetitive region 2; NJ: Neighbor-joining; nt: nucleotide; R: repetitive region; rDNA: ribosomal DNA; rRNA: ribosomal RNA

## Authors' contributions

CDA collected the sequence data, performed the statistical analyses and drafted the manuscript. TJC conceived of the study, participated in its design and coordination and helped to draft the manuscript. Both authors read and approved the final manuscript.

## Supplementary Material

Additional file 1 **Structure of complete IGS sequences**. PDF file showing features of 13 IGS sequences from 4 species in the *Daphnia pulex *complex. Click here for file

Additional file 2 **Gene conversion analysis of complete IGS sequences**. PDF file showing results of a gene conversion analysis of complete IGS sequences from 4 species in the *Daphnia pulex *complex using GENECONV. Click here for file

Additional file 3 **Gene conversion analysis of the IGS N1 region**. PDF file showing results of a gene conversion analysis of IGS N1 sequences from 4 species in the *Daphnia pulex *complex using GENECONV. Click here for file

Additional file 4 **Gene conversion analysis of the IGS N2 region**. PDF file showing results of a gene conversion analysis of IGS N2 sequences from 4 species in the *Daphnia pulex *complex using GENECONV. Click here for file

Additional file 5 **Gene conversion analysis of the IGS A repeat region**. PDF file showing results of a gene conversion analysis of IGS A repeat region from 4 species in the *Daphnia pulex *complex using GENECONV. Click here for file

Additional file 6 **Gene conversion analysis of the IGS B repeat region**. PDF file showing results of a gene conversion analysis of IGS B repeat region from 4 species in the *Daphnia pulex *complex using GENECONV. Click here for file

Additional file 7 **Gene conversion analysis of the IGS C repeat region**. PDF file showing results of a gene conversion analysis of IGS C repeat region from 4 species in the *Daphnia pulex *complex using GENECONV. Click here for file
