# Supplementary material for: Evolution of the nuclear ribosomal DNA intergenic spacer in four species of the Daphnia pulex complex
Source: BMC Genet. 2011 Jan 24;12:13. doi: 10.1186/1471-2156-12-13 (PMC3036644; doi:10.1186/1471-2156-12-13)
Supplement: Additional file 1 — Structure of complete IGS sequences. PDF file showing features of 13 IGS sequences from 4 species in the Daphnia pulex complex. [file 1471-2156-12-13-S1.PDF]

Additional file 1. Structure of complete IGS sequences from ten individuals representing four species in the *Daphnia pulex* complex. DpxNA = *D. pulex* North America, DpxE = *D. pulex* Europe, Dpc = *D. pulicaria*. Dten = *D. tenebrosa*. Length in in nucleotides.

IGS Sequence	IGS Length	%G+C	%A+T	N1 Length	A repeat No. of copies	A repeat Mean length	B repeat No. of copies	B repeat Mean length	C repeat No. of copies	C repeat Mean length	N2 Length
Dten	5072	46.19%	53.81%	769	4	214.3	4	99.75	2	192	2663
DpxNA1	4987	45.78%	54.22%	660	4	213.8	4	100	2	192	2688
DpxNA2	5174	45.57%	54.39%	660	5	210.2	4	99.75	2	192	2680
DpxNA3	4977	45.71%	54.29%	659	4	214.5	4	100	2	192	2676
Dpc1	4293	46.96%	52.99%	797	1	222	2	96	2	192	2698
Dpc2	4872	46.69%	53.29%	778	4	184	3	98.7	2	192	2678
Dpc3	5090	46.10%	53.90%	762	4	214.3	4	99.75	2	192.5	2687
DpxE1a	4761	46.68%	53.30%	765	3	212	3	98.7	2	192	2680
DpxE1b	4903	46.10%	53.90%	770	4	214.5	4	99.75	2	192	2492
DpxE2a	4770	46.16%	53.84%	763	3	213.7	3	98.3	2	192	2687
DpxE2b	4776	46.69%	53.31%	763	3	213	3	99	2	192	2693
DpxE3a	4992	46.88%	53.13%	765	3	197.7	6	99.5	1	194	2843
DpxE3b	4863	46.55%	53.45%	778	3	221.3	4	98.5	1	184	2843
