# Supplementary material for: Evolution of the nuclear ribosomal DNA intergenic spacer in four species of the Daphnia pulex complex
Source: BMC Genet. 2011 Jan 24;12:13. doi: 10.1186/1471-2156-12-13 (PMC3036644; doi:10.1186/1471-2156-12-13)
Supplement: Additional file 2 — Gene conversion analysis of complete IGS sequences. PDF file showing results of a gene conversion analysis of complete IGS sequences from 4 species in the Daphnia pulex complex using GENECONV. [file 1471-2156-12-13-S2.PDF]

Additional File 2. Putative gene conversion tracts between pairs of 13 complete IGS sequences from four species of the *Daphnia pulex* complex. Repeat arrays were replaced with one copy of the consensus for each repeat type. Tracts were computed in GENECONV v. 1.81. BC = Bonferroni corrected. KA = Karlin-Altschul. Num Poly = the number of polymorphic sites within the fragment. Tot Difs = the total number of sites at which the two sequences differ.

Global inner fragments	Simulated P-value	BC KA P-value	Aligned Begin	Offsets End	Length	Num Poly	Tot Difs
DpxE1b;DpxE1a	0.0342	0.10863	1846	3465	1620	92	37
Dten;Dpc2	0.0353	0.11108	3038	3453	416	42	81
DpxE1b;Dpc2	0.0283	0.08853	3871	4575	705	53	66
Dten;Dpc2	0.0008	0.00632	3871	4575	705	53	81
DpxE1a;Dpc2	0.0314	0.0986	3917	4591	675	48	72
DpxE3b;DpxE3a	0.0297	0.09123	4188	4549	362	33	103
DpxE1b;DpxE2a	0.0001	0.00041	4228	4989	762	71	73
DpxE1b;DpxE2b	0.0014	0.00856	4228	4701	474	48	87
Dten;DpxE2b	0.033	0.10306	4228	4586	359	33	102
DpxNA3;DpxE2b	0.0305	0.09634	4323	4701	379	42	82
DpxE1a;DpxE3a	0.0483	0.15126	4577	4682	106	11	231
DpxE3b;DpxE3a	0.0107	0.03242	4590	4989	400	36	103
**Additional pairwise fragments**							
DpxE1b;DpxE1a	0.0078	0.10863	1846	3465	1620	92	37
**Global outer fragments**							
Dten	0	0	4590	4671	82	8	335
Dten	0.005	0.02941	2776	2866	91	4	335
DpxE3a	0.0395	0.15841	1	701	701	6	287
