# Supplementary material for: Evolution of the nuclear ribosomal DNA intergenic spacer in four species of the Daphnia pulex complex
Source: BMC Genet. 2011 Jan 24;12:13. doi: 10.1186/1471-2156-12-13 (PMC3036644; doi:10.1186/1471-2156-12-13)
Supplement: Additional file 3 — Gene conversion analysis of the IGS N1 region. PDF file showing results of a gene conversion analysis of IGS N1 sequences from 4 species in the Daphnia pulex complex using GENECONV. [file 1471-2156-12-13-S3.PDF]

Additional file 3. Putative gene conversion tracts between pairs of the N1 sequences in 13 IGS sequences from four species of the *Daphnia pulex* complex were computed in GENECONV v. 1.81. BC = Bonferroni corrected. KA = Karlin-Altschul. Num Poly = the number of polymorphic sites within the fragment. Tot Difs = the total number of sites at which the two sequences differ.

Global inner fragments	Simulated P-value	BC KA P-value	Aligned Begin	Offsets End	Length	Num Poly	Tot Difs
DpxNA3;DpxE3a	0.0097	0.21643	400	452	53	14	38
DpxNA1;DpxE3a	0.0317	0.41243	400	452	53	14	36
**Global outer fragments**							
DpxE3a	0.0032	0.05199	63	239	177	9	50
