# Supplementary material for: Evolution of the nuclear ribosomal DNA intergenic spacer in four species of the Daphnia pulex complex
Source: BMC Genet. 2011 Jan 24;12:13. doi: 10.1186/1471-2156-12-13 (PMC3036644; doi:10.1186/1471-2156-12-13)
Supplement: Additional file 4 — Gene conversion analysis of the IGS N2 region. PDF file showing results of a gene conversion analysis of IGS N2 sequences from 4 species in the Daphnia pulex complex using GENECONV. [file 1471-2156-12-13-S4.PDF]

Additional file 4. Putative gene conversion tracts between pairs of the N2 sequences in 13 IGS sequences from four species of the *Daphnia pulex* complex were computed in GENECONV v. 1.81. BC = Bonferroni corrected. KA = Karlin-Altschul. Num Poly = the number of polymorphic sites within the fragment. Tot Difs = the total number of sites at which the two sequences differ.

Global inner fragments	Simulated P-value	BC KA P-value	Aligned Begin	Offsets End	Length	Num Poly	Tot Difs
Dten; Dpc2	0.0006	0.006	2056	2780	725	62	41
DpxE1a; DpxE3a	0.0071	0.06923	2782	2887	106	11	142
Dten; DpxE3a	0.0246	0.17032	1306	1398	93	9	152
Dten; DpxE2b	0.0294	0.20625	2426	2791	366	37	50
Dten; Dpc2	0.0426	0.27538	1232	1649	418	44	41
DpxE1b; DpxE2b	0.0499	0.32758	2426	2909	484	52	34
**Additional pairwise fragments**							
DpxE2a; DpxE2b	0.0312	0.89213	1502	2909	1408	105	14
**Global outer fragments**							
Dten	0	0	2795	2876	82	8	197
Dten	0.0009	0.0081	968	1059	92	5	197
DpxE3a	0.0009	0.0081	1593	1635	43	5	197
Dpc1	0.0229	0.12691	2714	2753	40	4	196
