# Supplementary material for: Evolution of the nuclear ribosomal DNA intergenic spacer in four species of the Daphnia pulex complex
Source: BMC Genet. 2011 Jan 24;12:13. doi: 10.1186/1471-2156-12-13 (PMC3036644; doi:10.1186/1471-2156-12-13)
Supplement: Additional file 5 — Gene conversion analysis of the IGS A repeat region. PDF file showing results of a gene conversion analysis of IGS A repeat region from 4 species in the Daphnia pulex complex using GENECONV. [file 1471-2156-12-13-S5.PDF]

Additional file 5. Putative gene conversion tracts between pairs of the A-repeat sequences in 13 IGS sequences from four species of the *Daphnia pulex* complex were computed in GENECONV v. 1.81. BC = Bonferroni corrected. KA = Karlin-Altschul. Num Poly = the number of polymorphic sites within the fragment. Tot Difs = the total number of sites at which the two sequences differ.

Global inner fragments	Simulated P-value	BC KA P-value	Aligned Begin	Offsets End	Length	Num Poly	Tot Difs
Dpc3-A1; DpxE3a-A2	0.0135	> 1.0	1	34	34	14	24
DpxNA3-A3; DpxNA1-A1	0.0226	> 1.0	19	51	33	18	19
DpxNA3-A3; DpxNA3-A1	0.0226	> 1.0	19	51	33	18	19
DpxNA3-A3; DpxE1b-A1	0.0226	> 1.0	19	51	33	18	19
DpxNA3-A3; DpxE1a-A1	0.0226	> 1.0	19	51	33	18	19
Dpc3-A1; DpxE3a-A3	0.0324	> 1.0	1	34	34	14	23
DpxNA3-A3; Dten-A4	0.0442	> 1.0	0	46	37	17	19
DpxNA3-A3; Dpc2-A4	0.0442	> 1.0	0	46	37	17	19
DpxNA3-A3; DpxE1a-A3	0.0442	> 1.0	0	46	37	17	19
DpxNA3-A3; DpxE1b-A4	0.0442	> 1.0	0	46	37	17	19
**Global outer fragments**							
Dpc2-A2	0.0174	0.73724	21	27	7	4	43
