# Supplementary material for: Evolution of the nuclear ribosomal DNA intergenic spacer in four species of the Daphnia pulex complex
Source: BMC Genet. 2011 Jan 24;12:13. doi: 10.1186/1471-2156-12-13 (PMC3036644; doi:10.1186/1471-2156-12-13)
Supplement: Additional file 7 — Gene conversion analysis of the IGS C repeat region. PDF file showing results of a gene conversion analysis of IGS C repeat region from 4 species in the Daphnia pulex complex using GENECONV. [file 1471-2156-12-13-S7.PDF]

Additional file 7. Putative gene conversion tracts between pairs of the C-repeat sequences in 13 IGS sequences from four species of the *Daphnia pulex* complex were computed in GENECONV v. 1.81. BC = Bonferroni corrected. KA = Karlin-Altschul. Num Poly = the number of polymorphic sites within the fragment. Tot Difs = the total number of sites at which the two sequences differ.

Global inner fragments	Simulated P-value	BC KA P-value	Aligned Begin	Offsets End	Length	Num Poly	Tot Difs
DpxE3aC1; Dpc2C2	0.0135	> 1.0	80	197	118	9	7
DpxE3aC1; DpxNA3C2	0.0135	> 1.0	80	197	118	9	7
